# Bridging Organizational Divides in Health Care: An Ecological View of Health Information Exchange

**DOI:** 10.2196/medinform.2510

**Published:** 2013-10-29

**Authors:** Kim M Unertl, Kevin B Johnson, Cynthia S Gadd, Nancy M Lorenzi

**Affiliations:** ^1^Vanderbilt Implementation Sciences LaboratoryDepartment of Biomedical InformaticsVanderbilt University School of MedicineNashville, TNUnited States

**Keywords:** health information systems, qualitative research, ethnography, community networks, information sharing, organizational models, information ecology

## Abstract

**Background:**

The fragmented nature of health care delivery in the United States leads to fragmented health information and impedes patient care continuity and safety. Technologies to support interorganizational health information exchange (HIE) are becoming more available. Understanding how HIE technology changes health care delivery and affects people and organizations is crucial to long-term successful implementation.

**Objective:**

Our study investigated the impacts of HIE technology on organizations, health care providers, and patients through a new, context-aware perspective, the Regional Health Information Ecology.

**Methods:**

We conducted more than 180 hours of direct observation, informal interviews during observation, and 9 formal semi-structured interviews. Data collection focused on workflow and information flow among health care team members and patients and on health care provider use of HIE technology.

**Results:**

We structured the data analysis around five primary information ecology components: system, locality, diversity, keystone species, and coevolution. Our study identified three main roles, or keystone species, involved in HIE: information consumers, information exchange facilitators, and information repositories. The HIE technology impacted patient care by allowing providers direct access to health information, reducing time to obtain health information, and increasing provider awareness of patient interactions with the health care system. Developing the infrastructure needed to support HIE technology also improved connections among information technology support groups at different health care organizations. Despite the potential of this type of technology to improve continuity of patient care, HIE technology adoption by health care providers was limited.

**Conclusions:**

To successfully build a HIE network, organizations had to shift perspectives from an ownership view of health data to a continuity of care perspective. To successfully integrate external health information into clinical work practices, health care providers had to move toward understanding potential contributions of external health information. Our study provides a foundation for future context-aware development and implementation of HIE technology. Integrating concepts from the Regional Health Information Ecology into design and implementation may lead to wider diffusion and adoption of HIE technology into clinical work.

## Introduction

### Background

Over 10 years ago, the Institute of Medicine identified the health care system of the United States to be “fragmented”, “distributed”, and “complex”. These attributes were viewed as challenging and potentially hazardous to the health care system [1,2]. Health care organizations have made significant progress in improving patient safety and moving toward patient-centered care over the last decade [3,4]. The current fragmented structure of health care delivery [5], however, directs patients to providers at multiple organizations for care [6,7], leading to dispersed and fragmented health information [8] and decreased continuity of care [9]. Care fragmentation impedes coordinated and cohesive health care delivery [1,2,9,10] and creates patient safety risks [3-5].

Health information technology solutions such as electronic health records can assist in reducing information fragmentation [5,11,12] within organizations, but solutions to share information across organizational boundaries are also needed [6,7,9,13]. Technology-supported [8,14-16] and federally funded [9,17] health information exchange (HIE) pilots are beginning to improve access to patient health information across organizational boundaries. Patients have also shown enthusiasm for this type of health information technology and accept that HIE can improve health care delivery [18]. Various approaches to interorganizational HIE have faced challenges due to disparate health information technology [19,20], organizational issues [21,22], and contextual factors related to workflow [23,24] and medical specialty [25]. Federal mandates requiring interoperability in health information technology design [26] have improved technological support for data exchange, but limited research has examined the direct impact of HIE on patients, health care providers, and the health care system as a whole.

We propose a new context-aware [27,28] perspective, the Regional Health Information Ecology, for examining the complex sociotechnical and organizational structures that emerge with successful implementation of HIE technology. Along with this perspective, our research question was how does the structure of a regional health care environment change when health information flows across organizational boundaries with technology support? Our goal in examining information exchange through this perspective was to investigate how technology and the health care system can coevolve to reduce information fragmentation and improve care coordination. While the setting for our study was a specific HIE technology implementation and a regional publicly funded HIE model, lessons learned relating to the Regional Health Information Ecology are widely applicable to different types of HIE technology at various design and implementation stages.

### Analytical Framework

A central tenet driving our research is that new analytical approaches are needed to examine the complex relationships involved in and generated by HIE technology projects. During initial fieldwork focused on HIE-related workflow, we observed interorganizational interactions that were evocative of ecology studies. After completing an extensive open-ended grounded theory analysis [29] of our qualitative ethnographic data [23], we applied the Information Ecology Framework [30] to provide structure for additional data analyses.

The Information Ecology Framework takes a sociotechnical approach [31] toward understanding interrelationships among people and technology in specific local settings. Nardi and O’Day described the information ecology concept as [30]:

A system of people, practices, values, and technologies in a particular local environment. In information ecologies, the spotlight is not on technology, but on human activities that are served by technology.

Five primary properties support application of the information ecology concept to a wide variety of environments (Table 1): system, locality, diversity, keystone species, and coevolution.

Researchers have applied the Information Ecology Framework to diverse contexts, including libraries [32,33], classrooms [34,35], computerized physician order entry [36], health care service delivery for homeless young people [37], surgical units in hospitals [38], virtual communities [39,40], and theme parks [41]. These scenarios share a common goal: to analyze data through the Information Ecology lens to better comprehend relationships among contextual elements.

Researchers working with large research datasets have proposed extending information ecology concepts to systematic levels and broader scales [42]. Rather than focusing only on interactions within a specific local ecology, this perspective extends the information ecology metaphor to interactions among ecologies [42]. Building on this idea, we examined HIE relationships by extending information ecology concepts and developing a new construct, the Regional Health Information Ecology. The Regional Health Information Ecology construct comprises multiple competing organizations with multiple clinical locations working together toward the common goal of information sharing, forming a dynamic exchange centered on health information.

**Table 1:** Information ecology components.

Component	Component characteristics
System	Interrelationships and dependencies among different parts of the ecology
Locality	Context in which technology is used including ownership of technology, networks around the technology, and connections related to the technology
Diversity	Niches for different roles and functions, different kinds of people and tools working together in a complementary fashion
Keystone species	Informal categories of people and tools necessary for the ecology to survive, based around informal rather than formal roles
Coevolution	Social and technical aspects of the ecology evolving together

## Methods

### Overview

The study design incorporated an iterative process of direct observation [43], semi-structured interviews [44], and data analysis to evaluate the impact of a Web-based HIE technology across widely varying clinical contexts, organizations, practice settings, and technology infrastructures. Our prior research into workflow, information flow, and technology use indicated that direct observation and semi-structured interviews were appropriate methods for the open-ended research questions motivating the study [45]. We previously discussed the setting, site selection, and data collection methods for the current study in great depth in a publication focused on workflow and HIE technology [23]. We provide a brief overview of data collection methods here and focus on the primary distinguishing characteristic of this portion of the larger study: applying a novel approach during data analysis using the Regional Health Information Ecology construct.

### Study Setting and Sampling Plan

The study setting was the MidSouth eHealth Alliance, a regional health information organization in Memphis, Tennessee [14]. The organization, also referred to as “eHealth” by HIE technology users, comprised the majority of health care organizations in the region. The exchange design used a “pull” approach [14]. Users logged into the HIE website, separate from their internal electronic health record. Depending on site-specific factors, users retrieved patient information using links based on the site’s recent patients registry or by entering identifying information for the patient such as name and date of birth. Data were retrieved based on a matching algorithm [46] and presented to the users in a list of matching documents. Patient data were included in the exchange unless a patient opted out of participation.

The primary HIE technology users during the study were health care providers in emergency department (ED) settings and in two major safety net [47] ambulatory care groups [48]. We designed a purposive sampling plan [49,50] to cover regional geographic zones, HIE technology usage levels, and both ED and ambulatory contexts. The health information technology infrastructure and use of HIE technology varied substantially across participating organizations and research sites [23,48]. The study took place before mandates requiring interoperability of electronic health records were enacted [51]. The Vanderbilt University Institutional Review Board and appropriate regulatory groups at each research site approved all study procedures. All data relating to specific participating organizations were anonymized throughout this report, at the request of participating organizations.

### Data Collection

#### Observation and Informal Interviews

One researcher (KMU) observed health care providers engaged in clinical work for over 180 hours, spread across 6 EDs and 8 ambulatory clinics spread through the Memphis region. Observation focused on interactions with the HIE technology and work practices to provide contextual details about HIE technology use. All primary observation subjects had HIE technology access, but actual technology use varied among subjects. The observer recorded detailed notes about technology use and work practices during observation. Throughout observation, the observer conducted informal interviews with observation subjects and other health care workers. The observer sought to be unobtrusive throughout data collection, avoiding disruption of routine work practices as much possible. The observer transcribed notes into an electronic notebook application [52] and later transferred these notes to NVivo 8 software [53] to organize qualitative data analysis.

#### Semi-Structured Interviews

A researcher (KMU) conducted nine semi-structured telephone interviews, after completing observation. When possible, we selected interview subjects who we also observed. To incorporate the widest range of perspectives on HIE, we also interviewed some subjects who were not observed (eg, health care providers with limited work schedules, medical directors, information technology managers). Interview questions explored information seeking behavior, information needs, impact of HIE technology use, and general feedback on HIE technology design and implementation. We designed the interview questions to provide member checking [54] of observation data analysis and to collect additional open-ended feedback about the HIE technology. We audiotaped the interviews, transcribed the interview recordings, and transferred the interview transcripts to NVivo 8 for data analysis. Interview subjects received a small gift card in appreciation of their time.

### Data Analysis: Axial Coding

Our initial approach to data analysis involved an open-coding grounded approach [29]. After completing this initial grounded approach to data analysis, we moved into framework-focused data analysis and an axial coding approach [55,56]. Our approach to data analysis first developed theory emerging from the data itself and later applied existing theoretical frameworks to our understanding of the data. For example, during open coding, codes related to reasons health care providers used the HIE technology and the outcomes of technology use emerged repeatedly across observation data. These codes coalesced into the themes “prompts for HIE use” and “outcomes of HIE use.”

The Information Ecology Framework provided structure during axial coding. We assessed all observation and interview data for elements of the Regional Health Information Ecology construct, using five Information Ecology elements (Table 1) to guide coding. During axial coding guided by the Information Ecology Framework, we focused on understanding roles involved in information exchange and informal and formal interorganizational connections. For example, codes related to individuals involved in information exchange identified the “keystone species” for information exchange. Based on axial coding analysis, we manually developed graphical models about inter- and intraorganizational connections before and after HIE technology availability using diagramming software [57]. The graphical models provided visual maps of the Regional Health Information Ecology construct.

### Confirmability

Our research employed a systematic and rigorous approach toward ensuring and evaluating credibility, transferability, and dependability [54]. We designed a multistage confirmability strategy with components during fieldwork, during data analysis, and after fieldwork.

We established credibility, analogous to internal validity [54], through three distinct processes: field research activities, peer debriefing, and member checking. Field research activities to establish credibility included prolonged engagement, persistent observation, and triangulation. We allotted lengthy periods of time in the project timeline to allow for prolonged engagement with and immersion in the environment [58]. We conducted data analysis concurrent with data collection, allowing emerging themes to provide depth and direction of data collection, meeting the purpose of persistent observation [59]. Triangulation strategies included using multiple sources of data and applying multiple methods [60]. Interaction with a peer debriefer and member checks were used throughout the research project as additional approaches to ensure credibility. A peer debriefer served as a “devil’s advocate” in discussing methodology, data analysis, and general fieldwork topics [54]. Member checking through informal and formal interviews consisted of discussing research findings with research subjects to collect additional layers of data, to gain feedback on the accuracy of the data, and to provide a different perspective on the findings [54].

Our emphasis for transferability, analogous to external validity [54], was on transferability of research findings to similar contexts. To facilitate this, we developed a rich description of findings and a thorough description of context to allow comparison of contextual similarities between different research sites. The project timeline allocated adequate time to investigate several distinct sites, providing evidence of transferability of the findings.

Dependability is analogous to reliability [54]. Throughout the project, the primary researcher engaged in activities to encourage reflexivity. Reflexivity involves being aware of the influence of the researcher’s perspective on the collection, interpretation, and analysis of the data [61]. Journaling allowed the researcher to record information such as personal reasons for selecting the research topic, perspectives on the research, reactions to fieldwork activities, and other information not appropriate in formal field or methodology notes. The process promoted awareness of potential sources of bias for the investigator and made the perspective of the researcher transparent to others. In addition, this process allowed the investigator to “bracket” sources of individual bias in an attempt to filter them from the research [62].

## Results

### Health Information Exchange

The core of HIE involves constantly shifting and evolving relationships among people, organizations, and technology. Prior to HIE technology availability, Memphis organizations and individuals exchanged health information manually through both formal and informal processes. Manual information exchange processes used approaches such as phone calls, faxing, and mail. Access to HIE technology automated portions of the formal level of data exchange. Information exchange processes remained fragmented, despite the HIE technology. Health care providers at all participating sites used manual information exchange processes in addition to technology-supported processes. Reasons that we observed for parallel manual and automated processes included amount and type of data available through the HIE technology, lack of HIE technology access, and limited technology use. Coexistence of manual and automated processes allowed us to examine HIE practices and the information ecology both with and without technology support for information exchange.

### Mapping Information Ecology Concepts on the Regional Level

#### Key Components

Based on observation and interview data, we mapped the five main Information Ecology Framework components to the Regional Health Information Ecology (Table 2).

**Table 2:** Key components of the Regional Health Information Ecology.

Component	Component characteristics
System	Multiple competing health care organizations in the region
	Multiple clinical sites within each organization
	Need for data exchange within organizations and among competitors to support continuity of patient care
	Transfers in responsibility for patient care among inpatient and outpatient environments
	Information flow mediated by patient involvement
Locality	Overall local region
	Health care community within the region
	Organizations within the health care community
	Individual sites within organizations
	Specific departments at each site (ie, ED, specialty clinic)
Diversity	Many formal and informal roles involved in information exchange:
	Patients and caregivers: report visits to other hospitals/clinics
	Physicians: ask nurses and administrative staff to obtain external records
	Resident physicians: informal sources of patient health information
	Nurses: obtain formal consent for information exchange from patients
	Administrative staff: collect records from other organizations
	Records clerks: locate records and fax to other organizations
Keystone species	Information consumers: nurses, nurse practitioners, physicians, individuals who need information from other sites as part of the medical decision-making process
	Information exchange facilitators: people with knowledge of who to contact at other organizations and of procedures/requirements of other organizations
	Information reservoirs, informal: resident physicians contacted by resident physicians at other locations, patients discussing visits to other hospitals
	Information reservoirs, formal: patients bringing medical records from other sites, information repositories such as electronic health records and paper charts
Coevolution	Constantly shifting process for obtaining health information, related to:
	Organizational policies
	Information repositories at different institutions
	Changes in staffing
	Resource shifts
	Technology availability and accessibility

#### Ecology Component: System

The Regional Health Information Ecology *system* consisted of multiple health care organizations with long-standing competitive relationships. Each organization comprised multiple clinical sites, including hospitals and ambulatory clinics. Shared health information technology infrastructures facilitated the flow of health information within each separate organization and provided access to patient records at multiple clinical locations. Prior to the HIE technology, each organization was in effect an information silo and did not share data with other organizations. One administrator described views of information in her organization by saying, “We were used to our information being in our control.” Patients moved between inpatient and outpatient environments and between different organizations, resulting in incomplete patient health data within any single organizational information silo. The patient mediated information flow prior to HIE technology, with health care team members seeking external information in response to a patient mentioning visits to other organizations.

#### Ecology Component: Locality

To capture the full extent of *locality* during data collection, we observed at sites across the region seeking to broadly represent local clinical environments. The sites represented multiple organizations and both ED and ambulatory environments. Each site represented a distinct local context and used different types of information repositories to store health information, ranging from paper charts to electronic health records. Until HIE technology implementation, a single unifying form of technology did not exist across all participating sites. Physicians in ED settings repeatedly described the patient population in the region as “mobile”, with one physician noting that “With all these hospitals in close proximity, patients tend to go where they think wait time is lowest at that time.”

#### Ecology Component: Diversity

Individuals in a variety of roles participated in HIE activities, exhibiting role *diversity*. Patients and their caregivers played key roles in information exchange, by reporting visits to other clinical sites and prompting physicians to seek out external health information. Members of the clinical care team including nurses, nurse practitioners, and physicians participated in information exchange to different degrees. Administrative staff and records clerks facilitated information exchange, particularly in the manual information exchange processes, where administrative staff faxed requests to other organizations and records clerks retrieved data.

#### Ecology Component: Keystone Species

We identified three function-based *keystone species* that formed the basis of HIE regardless of HIE technology presence: information consumers, information reservoirs, and information exchange facilitators.

Information consumers needed and sought information from external sites for a variety of reasons. Providers in the ambulatory care environment required information from hospitals or referral sites their patients visited to ensure continuity of patient care and to provide data for use in medical decision making. Ambulatory, ED, and inpatient care providers sought external medical detail to learn about already-completed diagnostic procedures and other general medical history details.

Information reservoirs come from many different perspectives and roles and have both formal and informal roles in storing health information. Based on official health information privacy regulations, medical records clerks served as the main formal information reservoirs. After receipt of appropriate patient authorization forms, medical records clerks working at a specific site retrieved patient data from their site’s electronic or paper-based information repositories and sent data to the requesting site. Multiple informal reservoirs of external information participated in information exchange, including patients, family and friends of patients, residents, and other providers. Patients provided a layperson’s perspective on details of care episodes at other sites, results of recent diagnostic procedures, and information on diagnoses. While this level of information was helpful, clinicians often required additional detail for medical decision making. One ambulatory physician described information from patients by saying,

I have a lot of patients who just don't seem to understand what happened to them or what they tell me just doesn't make sense. So, I go to eHealth to clarify those sorts of questions. If they don’t seem to be able… they don’t seem to understand what happened to them. Or what they were told.

The gap between a layperson’s description and the level of information needed for clinical decision making was also a problem in the ED, as a physician described,

Sometimes, the patients that we see in the emergency room don’t solely come to [my hospital] for their care, and they have been to other hospitals in the Memphis area and had tests done or lab work drawn, x-rays, EKGs, etc and they don’t know the full extent of the results of those tests. They can tell us that they had them done and if the doctor said that something was wrong... but they can’t give us the detail that we need with which to treat them on that particular day and so, that’s one way that eHealth helps us a lot because we’re able to pull up results from most of the other hospitals in the Memphis area and see exactly what they’re trying to explain to us in laymen’s terms and help us determine what studies to gear our workup for that particular day.

During observation, we also identified another informal information reservoir: resident physicians. Residents affiliated with an academic medical program in the region formed an informal communications network as they rotated through different organizations in the region, providing them with access to different information repositories. This informal route often provided faster access to data than formal information exchange processes. Residents were both information reservoirs and information consumers in this instance.

Information exchange facilitators bridged the gap between information consumers and information reservoirs by assisting in intersite information transfer. Prior to HIE technology availability, a variety of groups filled this role: referral clerks, medical records clerks, registrars, other administrative staff, and also medical staff including nurses and physicians.

#### Ecology Component: Coevolution

The final information ecology concept that we mapped to the Regional Health Information Ecology was *coevolution*, the evolution of technology and individual work practices together over time. Both manual and technology-supported HIE processes were constantly shifting due to multiple factors. The individuals participating in information exchange constantly shifted due to changes in medical, administrative, and information technology staffing. Organizational policies toward HIE technology evolved over time, resulting in changes to what roles had technology access and who was responsible for supporting the technology. For example, one organization initially selected administrative clerks for HIE technology access, but over time moved access to nurse practitioners and physicians. The HIE technology continued to change in response to shifting organizational and user requests. The overall process of HIE and the technology supporting the process coevolved over time.

### Impact of HIE Technology on the Information Ecology

#### HIE Technology Implementation

**Figure 1:**
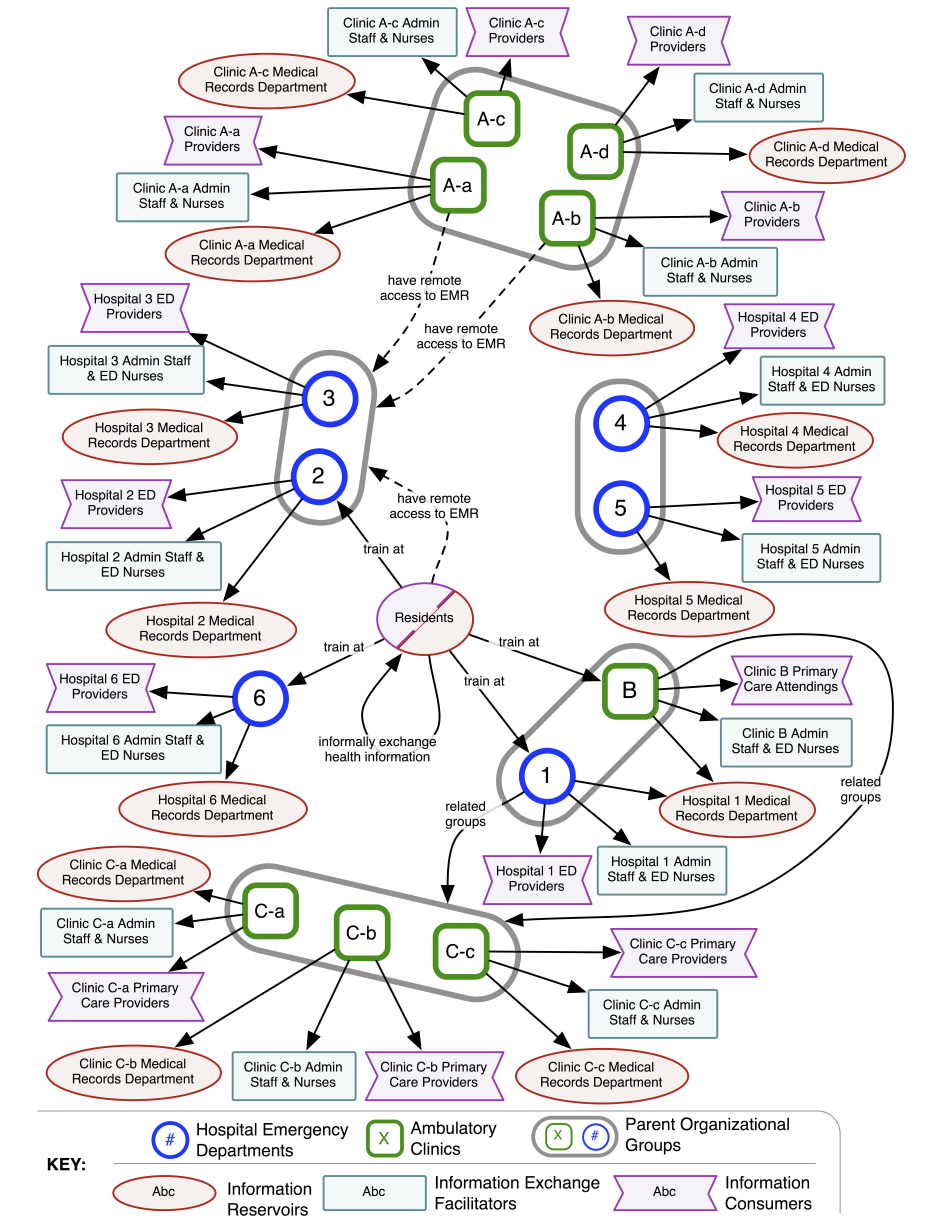
Regional Health Information Ecology, before HIE technology implementation.

Figure 1 summarizes the Regional Health Information Ecology before HIE technology implementation. Information silos characterized the Regional Health Information Ecology, with limited health information availability outside of each parent organization. Information consumers such as physicians and nurse practitioners followed a formal and manual process to obtain data from external health care organizations. Information exchange facilitators including administrative staff and nurses bridged gaps between organizations, using fax machines and phone calls to convey information requests. Information repositories within medical records departments at each organization controlled external access to health information. An informal data exchange process facilitated by informal connections among resident physicians coexisted with the formal manual data exchange process.

**Figure 2:**
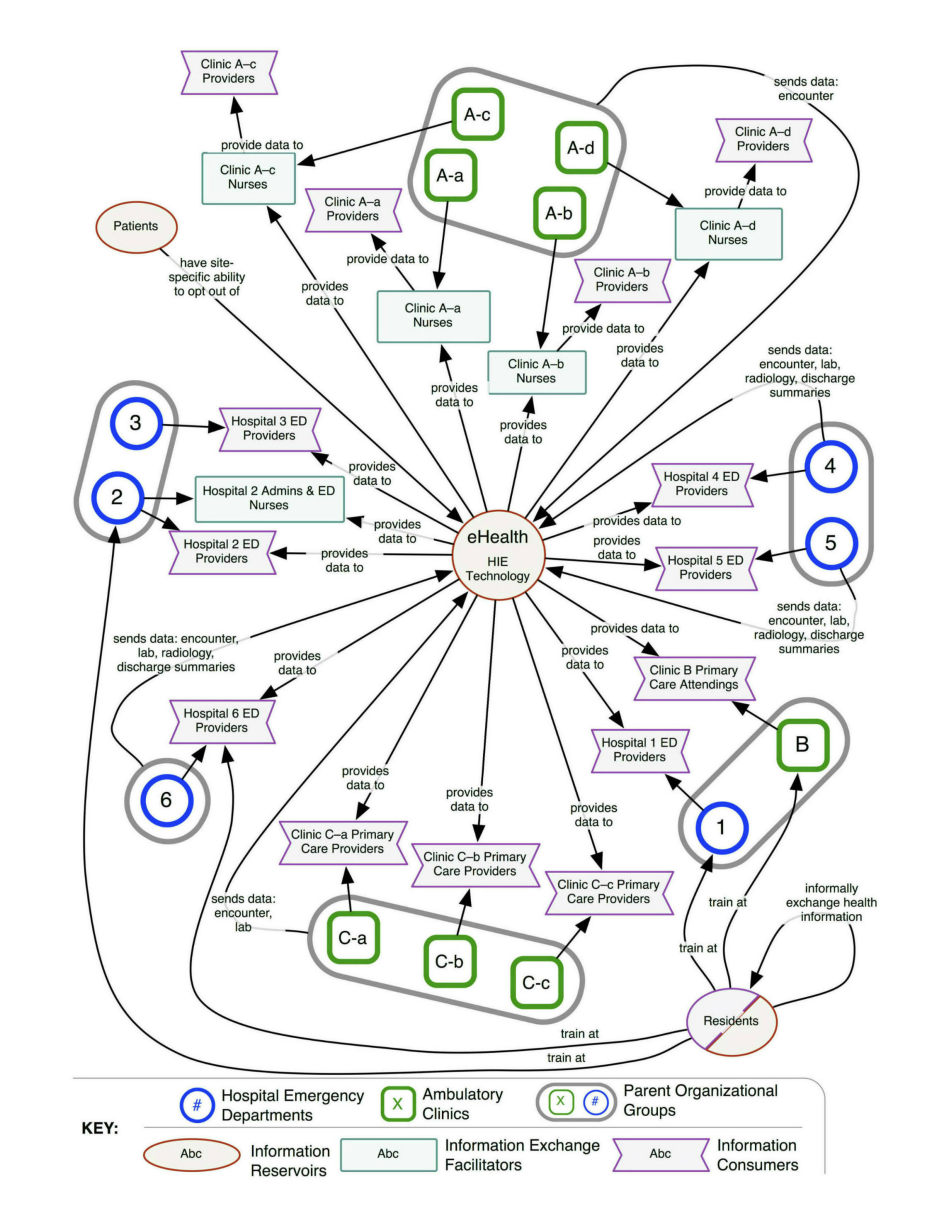
Regional Health Information Ecology, after HIE technology implementation.

HIE technology created a centralized information resource. Automated approaches to information exchange shifted roles and responsibilities and created new forms of interorganizational connections (Figure 2). The technology-supported information exchange process substantially changed how organizations exchanged information. Information consumers were able to directly access external health information. The roles of information exchange facilitators and information repositories were minimized in the new automated approaches. Availability of HIE technology fundamentally altered the Regional Health Information Ecology.

However, limited access to and adoption of the HIE technology resulted in both manual and automated information exchange processes coexisting. Availability of HIE technology did not fully replace the manual information exchange process. The Regional Health Information Ecology structures shown in Figures 1 and 2 both existed after HIE technology implementation. An overview of the study and its results can be found in Multimedia Appendix 1.

We identified three categories of changes to the Regional Health Information Ecology with the introduction of HIE technology:

1. Moving from health information fragmentation toward unification, characterized by a shift from separate information silos and information fragmentation toward a more cohesive view of patient health.
2. Reduced time to obtain health information, created by streamlined processes to access information and a reduction in the role of information exchange facilitators.
3. Improved interorganizational communication among information technology departments, indicated by a shared sense of knowledge about health information technology practices in the community.

We will discuss each of the three categories in detail.

#### Moving From Health Information Fragmentation Toward Unification

Observation of both technology-supported and manual HIE processes illustrated the highly fragmented nature of each patient’s individual health information. Even in a hypothetical scenario where a patient only visited clinics and hospitals sharing common information repositories, some external health transactions like filling prescriptions would occur outside that shared information environment. Observed patient situations were far more complex than this hypothetical scenario. Patients visited multiple clinical sites and health care organizations for primary and specialty care. We also observed a lack of intraorganizational information exchange within some organizations. In one organization, electronic health record systems used in their hospital and their ambulatory clinic group did not share data, introducing difficulty in following up after patients were discharged from the hospital. In other organizations, the ED information system was unable to share data with the hospital’s electronic health record, creating information gaps when patients moved from the ED to inpatient care. Paper-based documentation processes further contributed to information fragmentation. Health care providers noted that the information available through HIE technology did not provide a complete record of all of the patient’s health care information, but that the HIE technology improved information availability. As one ED physician stated,

For the first time, we’ve become more like a doctor in an office type practice, where we have that sort of continuity of care information that’s never been available. It’ll never be on par with a doctor’s office, but we’re getting a whole lot closer.

#### Reduced Time to Obtain Health Information

Manual information exchange processes resulted in delays of information availability, described by providers as waiting for “hours” or “days” to receive requested records and sometimes never receiving them. Information exchange delays were particularly challenging in the ED setting. One ED physician described these challenges by saying,

Before, if I was working during the day, I could at least still contact the medical records department of another facility, get the patient to sign a consent form, fax the information over, and then hope that someone would fax it back to me. That wasn't always foolproof, but at least during the day, there was some chance of it happening. But if I worked the evening or overnight shift, I was just frustrated, because most medical records departments aren’t open for outside help overnight.

Delays in information availability affected not just providers but also patients as noted by one technology manager,

We’d been waiting for like three hours for one of the hospitals to fax over discharge summaries and stuff for somebody who was referred. As soon as [the providers] looked into eHealth they could get all that information right then and the patient didn’t have to sit in the waiting room for another hour or two. They could be seen, right then and there.

Availability of HIE technology increased the ability of health care providers to access health information, regardless of day or time, and had potential to improve patient health care experiences. The HIE technology also impacted medical decision making, by making information available immediately. One ambulatory physician noted, “Now, I can make a decision… having access to tests right now makes a big difference as far as making choices at the bedside.” An ED physician described the impact of information obtained through the HIE technology, “Everything you do, it helps narrow the field on what you’re having to deal with.”

#### Increased Provider Awareness of Patient-Health System Interactions

Our previous grounded analysis of our data explored trust-related use of the HIE technology and provider use of HIE technology to identify individuals seeking narcotic medications [23]. Use of the HIE technology also increased provider awareness of patient interactions with other health care organizations. For example, ED providers could quickly identify if patients had a recent visit to another ED in the region. Knowledge of patient visits to other hospitals and procedures performed elsewhere changed how providers moved forward with medical decisions, as described by one ED physician,

Again, oftentimes it will just be that they just were at another hospital and had this complete workup done; they didn’t share that with you. When you confront them with that, “Hey, you just left [another hospital], what did they tell you?” Then it just totally changes, changes what you do.

Another ED physician discussed the impact of HIE technology on continuity of care,

The only thing we had before eHealth was sort of the continuity of the same doctors at the same place and as you kind of got to know patients a lot of times you can root some of this [information] out, but this helps earlier in the process now, you don’t have to wait until you have some kind of personal experience with them. You’ve expanded your personal experience with them.

ED providers also used the HIE technology to identify individuals possibly using the ED for primary care and attempt to redirect those individuals to ambulatory care resources, as described by one ED physician,

With frequent ER visits, I’m looking to see if they use the ER instead of going to clinics, use the ER for minor health issues, so that I can encourage them to find a primary care physician and maybe try to hook them up with a clinic they can use.

The HIE technology also assisted primary care physicians with understanding recent hospitalizations or other health care interactions. The type of information available through the HIE technology provided greater depth than patient-provided information. An ambulatory physician described one patient scenario,

I had a patient who was hospitalized and when I checked the record [in eHealth], the discharge summary mentioned they had HIV as part of their diagnosis and they just didn’t tell me that. They had fifteen other things that went wrong and when I called her, she said “Oh, yeah, I remember that… I just forgot and didn’t mention it.”

#### Improved Interorganizational Communication Among Information Technology Departments

HIE technology also impacted health information technology specialists in the region. Implementing and supporting the technology required communication among information technology (IT) support groups across organizations. Working with IT support at different organizations allowed communication that previously was not common in the region. One IT manager described this shift in thinking about IT support by saying,

It’s too bad that not every community, especially for IT, people at my level, to be able to talk to other people in real life, you’re not alone… so that even if our best practices aren’t industrial best practices, we can say that they’re regional health care IT best practices.

Implementation of the HIE technology fundamentally shifted how health care providers and IT support staff viewed other organizations in the region. Patient involvement in the design and implementation of the HIE technology was, however, quite limited. Patients were less aware than providers of the evolving process for information exchange among organizations, resulting in surprise for some patients when providers had access to information they had not disclosed.

### The Paradox of Nonuse and the Regional Health Information Ecology

Regardless of how frequently an individual used the HIE technology, health care providers uniformly described HIE as a useful contribution to health care. An ED physician who frequently used the HIE technology expressed how much she valued the system for providing patient care:

I think eHealth is quite useful to me. eHealth to my ability to treat patients is like a cell phone is to now. You know, if you look back, you say “How did I ever survive without a cell phone?” but somehow we managed to do it. It’s like now with eHealth, “How did I ever take care of patients without eHealth?” It has made a big difference.

Even providers who were only sporadic HIE technology users uniformly described how useful the technology was, with comments like “When we use it, it’s great.”

Integration of the HIE technology into health care practices varied across sites and among providers. Substantial inter- and intrasite usage variability presents an intriguing paradox that researchers previously described with other types of health information technology [63]. If providers value HIE technology, why was it used so infrequently? How does this paradox of nonuse impact the evolution and future of the Regional Health Information Ecology?

Previous research examined questions of HIE technology use and nonuse through quantitative approaches [64,65]; our research adds a complementary rich layer of description to the understanding of usage questions through qualitative approaches.

Inconsistencies in the amount of information and the types of information available through the HIE technology created a barrier to use. Participating organizations determined information sharing policies for their own organization. Some organizations provided both raw data (eg, laboratory reports) and summary data (eg, discharge summaries), while others provided only raw data or demographic data. Although some providers expressed concerns about information overload, more commonly providers stated that there was too little information available through the HIE technology. Providers identified discharge summaries as especially important, with one provider stating “Accessing labs and radiology is nice, but pulling up the discharge summary is the cherry on the cake.”

The HIE technology evolved over time as more hospitals and clinics contributed data and as the types of available data increased. For example, the HIE technology was initially directed only at EDs, but rapidly expanded to ambulatory clinics. The amount of data shared by ambulatory clinics was limited however, due to technology infrastructure barriers and ongoing organizational change. Despite efforts to communicate the availability of additional data, providers often seemed unclear on what data were available. Some providers, frustrated by initial data limitations early during implementation, stopped using the HIE technology altogether. Widely varying implementations of the HIE technology across organizations and specific sites increased the difficulty of reaching these providers.

Although providers uniformly discussed HIE technology as useful in general, some providers indicated it was not useful for their specific role. One ED provider stated, “I need to concentrate on life-threating illnesses. I don’t have time to go looking through the chart looking for records.” The same time pressure prevented some providers who used the HIE technology from understanding the full functionality of the tool. According to one ED provider,

I’m not sure if I’m using eHealth to its full potential. I’ve got in my little rut that I go through just because of repetition. Whether there’s a lot more to offer from it, I don’t know… I guess if I sat down and played with it, but I’m usually on it literally a few seconds at a time or a minute at a time and then I turn it off. Maybe there’s some unlocked potential there that I’m not even aware exists.

Our research also identified missed opportunities related to participating organizations and potential users for HIE technology. Although the majority of hospitals in the region participated in the HIE, two hospitals did not, causing gaps in information availability. During data collection, we identified several different types of health care sites outside of ambulatory and ED environments that could benefit from HIE participating including assisting living and nursing home facilities, radiology centers, and specialty clinics.

Organizations determined who had HIE technology access within their organization. We observed multiple cases of sharing logins and looking information up for other providers without HIE technology access, indicating a potential need for broader access. Groups that did not have HIE technology access that could benefit based on observation and interviews included: nurses, resident physicians, hospitalists, specialty care providers, and pharmacists. Nurses at several sites had HIE technology access, but not at all sites.

Our research identified a complex interplay of factors contributing to the paradox of nonuse of HIE technology in this specific Regional Health Information Ecology. The impact of addressing one or more of the factors identified through our research as contributing to nonuse requires changes in policy, technology, and organizations and concomitant evaluation.

## Discussion

### Principal Findings

The exchange of health information is integral to health care delivery, but significant gaps in information availability present a long-standing and continuing challenge. The manual and formal information exchange processes that we observed resulted from a culture of “information silos”, where organizations tightly controlled access to their own data and health care providers had limited expectations of data availability. Our study demonstrated that HIE technology opened these “information silos” by bridging information gaps among competing organizations. When used, HIE technology allowed providers to directly access health data across contexts and organizations. Direct data access reduced frustration caused by restricted access to external information and improved interorganizational information flow. The new form of information availability resulting from HIE technology allowed providers to proactively seek patient health information, rather than relying primarily on patient self-reports.

The changes in information flow that we observed demonstrated how the Regional Health Information Ecology evolved in response to technology-supported data exchange. Health care providers at some sites significantly altered their health information practices and expectations of data access. Inconsistent adoption levels within and across organizations, however, resulted in many sites where health information practices of health care providers remained effectively unchanged. Limited adoption of the HIE technology revealed gaps in how HIE technology designers and implementers viewed work practices related to information exchange.

Our research also demonstrated how provider perspectives about health information from outside their organization affected HIE technology adoption. Perspectives on health information needed to shift for successful HIE technology implementation and adoption. From a management and technology support perspective, HIE technology requires organizations to relinquish proprietary interest in health data. HIE technology challenges how health care providers view information, broadening the scope of information available for medical decision making. Approaches that assist with changing organizational perspectives on health information ownership are needed. Support for demonstrating to health care providers how this broader information base can contribute to patient care may also improve adoption. Based on observation and interviews, successfully implementing HIE technology requires awareness of perspective shifts required of health care providers.

Based on our research, we hypothesize that greater evolution of information exchange across a Regional Health Information Ecology requires consistently higher rates of HIE technology adoption and inclusion of a broader range of health care organizations across the region, suggesting foci for future HIE technology efforts. Our research revealed barriers to and opportunities for continuing evolution of the Regional Health Information Ecology. Strategies to extend the reach of the HIE technology could include adding more types of health care organizations to the exchange and working with organizations to provide HIE technology access to more health care roles. Existing progress with the information ecology demonstrates, however, the challenges of communicating dynamic system information across organizations. Strategies are needed to overcome barriers to improving HIE technology support for the Regional Health Information Ecology.

Future interorganizational data exchange efforts can build on this research; organizers should examine the Regional Health Information Ecology during HIE technology design and implementation. During initial design stages, organizers could apply awareness of the existing interorganizational landscape and navigate challenges created by long-standing competitive relationships. Implementation planners could use information ecology knowledge to develop an evidence-based approach to implementing exchange technology across organizations, sites, and clinical contexts. System designers could also tailor HIE technology to specific components of local contexts, to best meet regional and local needs. For example, understanding the role of informal information exchange processes, like the residents in the Memphis case, could provide a basis for design of technology features to support different perspectives on health information. Awareness of components of the Regional Health Information Ecology could also help HIE technology implementers to identify potential challenges prior to implementation. Our research suggests the need for HIE technology efforts to provide cross-organizational expertise to help guide technology implementation in specific contexts. While leaders within organizations know their context well, they may have limited experience with identifying challenges to achieving widespread technology adoption and use. Most importantly, lessons about information ecology and applications of HIE technology across organizations can provide an evidence base for context-specific design and implementation strategies.

### Limitations

Our research followed a rigorous protocol directed at ensuring confirmability of this qualitative research, but the study has several limitations. The research setting was one example of the application of a specific HIE technology approach to a specific regional context. We would expect specific details of a Regional Health Information Ecology to vary depending on multiple contextual factors, but the broader ecology concepts developed through this research are transferable to other settings and other HIE structures. A single researcher collected the data for this study, introducing the potential for observer bias. As with any observational study, the Hawthorne effect was a potential data confounder. Both the HIE technology and the regional health care environment changed during the course of the study. We addressed these potential limitations through multiple approaches. The data, data analysis processes, and outcomes of data analysis were discussed extensively with other members of the research team, formal and informal advisors specializing in ethnographic approaches, and a peer reviewer with extensive qualitative experience. Interviews with observation subjects and other exchange users provided member checking of observation analysis. The researcher asked probing questions throughout observation and interviews specifically directed at determining potential observer effects on work activities and potential researcher effects were carefully examined during data analysis. We incorporated information about changes to the HIE technology and to the regional health care environment in our data. We sought to address potential limitations of the study through these multiple approaches.

### Conclusions

The goal of our research was to examine the impact of HIE technology on the Regional Health Information Ecology as part of developing an in-depth understanding of the context. The context in this case was the MidSouth eHealth Alliance and the Memphis region. The research introduced a qualitative and ethnographic perspective to the evaluation of technology-supported HIE. By applying the Information Ecology Framework to HIE, we moved beyond the local interaction level and captured a spectrum of the ecology from the highly localized and individualized level of detail to the broader community level. Our study demonstrated that the Regional Health Information Ecology is a complex, constantly evolving Web of relationships among organizations and individuals.

Examining the Regional Health Information Ecology provides a foundation for future HIE efforts and a pathway toward customization of HIE systems. Although contextual factors vary among HIE implementation environments, the patterns of HIE use identified through this research and the methodology we applied can serve as a starting point for design and implementation efforts elsewhere. Other HIE structures such as smaller private exchanges or directed exchange could use the Regional Health Information Ecology concepts as a starting point for analysis of their own specific ecological constructs and needs. The next phases of interorganizational data exchange must build greater awareness of the needs and perspectives of intended HIE system users to achieve wider technology diffusion and adoption.

## Abbreviations

ED: emergency department
HIE: health information exchange
IT: information technology

## Multimedia Appendix 1

Study and results overview.
